# Kidney GATA3^+^ regulatory T cells play roles in the convalescence stage after antibody-mediated renal injury

**DOI:** 10.1038/s41423-020-00547-x

**Published:** 2020-09-11

**Authors:** Ryota Sakai, Minako Ito, Kyoko Komai, Mana Iizuka-Koga, Kazuhiko Matsuo, Takashi Nakayama, Osamu Yoshie, Koichi Amano, Hiroshi Nishimasu, Osamu Nureki, Masato Kubo, Akihiko Yoshimura

**Affiliations:** 1 Department of Microbiology and Immunology, Keio University School of Medicine, 35 Shinanomachi, Shinjuku-ku, Tokyo 160-8582 Japan; 2 Department of Rheumatology and Clinical Immunology, Saitama Medical Center, Saitama Medical University, 1981 Kamoda, Kawagoe, 350-8550 Japan; 3 Medical Institute of Bioregulation Kyushu University, 3-1-1 Maidashi, Higashi-ku, Fukuoka 812-8582 Japan; 4 Division of Chemotherapy, Kindai University Faculty of Pharmacy, Higashi-Osaka, 577-8502 Japan; 5 The Health and Kampo Institute, Sendai, Miyagi 981-3205 Japan; 6 Department of Biological Science, Graduate School of Science, The University of Tokyo, 7-3-1 Hongo, Bunkyo-ku, Tokyo 113-0033 Japan; 7 Center for Animal Disease Models, Research Institute for Biomedical Science, Tokyo University of Science, 2669 Yamazaki, Noda-shi, Chiba 278-0022 Japan

**Keywords:** Tissue Tregs, autoantibody, GATA3, kidney, IL-33, Regulatory T cells, Peripheral tolerance

## Abstract

FoxP3^+^ regulatory T cells (Tregs) play crucial roles in peripheral immune tolerance. In addition, Tregs that reside or accumulate in nonlymphoid tissues, called tissue Tregs, exhibit tissue-specific functions and contribute to the maintenance of tissue homeostasis and repair. In an experimental mouse model of crescentic glomerulonephritis induced by an anti-glomerular basement membrane antibody, Tregs started to accumulate in the kidney on day 10 of disease onset and remained at high levels (~30–35% of CD4^+^ T cells) during the late stage (days 21–90), which correlated with stable disease control. Treg depletion on day 21 resulted in the relapse of renal dysfunction and an increase in Th1 cells, suggesting that Tregs are essential for disease control during the convalescence stage. The Tregs that accumulated in the kidney showed tissue Treg phenotypes, including high expression of GATA3, ST2 (the IL33 receptor subunit), amphiregulin (Areg), and PPARγ. Although T-bet^+^ Tregs and RORγt^+^ Tregs were observed in the kidney, GATA3^+^ Tregs were predominant during the convalescence stage, and a PPARγ agonist enhanced the accumulation of GATA3^+^ Tregs in the kidney. To understand the function of specific genes in kidney Tregs, we developed a novel T cell transfer system to T cell-deficient mice. This experiment demonstrates that ST2, Areg, and CCR4 in Tregs play important roles in the accumulation of GATA3^+^ Tregs in the kidney and in the amelioration of renal injury. Our data suggest that GATA3 is important for the recruitment of Tregs into the kidney, which is necessary for convalescence after renal tissue destruction.

## Introduction

Regulatory T cells (Tregs) play crucial roles in peripheral tolerance and tissue homeostasis in the immune system.^1–3^ They express FoxP3 as a major master transcription factor and suppress excessive immune responses by recognizing various self and foreign antigens. In addition to immune suppression functions, Tregs residing in various nonlymphoid tissues, such as fat, skin, lungs, and intestines, at a steady state, termed tissue Tregs, have been shown to play essential roles in tissue homeostasis and to repair tissues via tissue-cell interactions.^4–6^ In addition, Tregs often accumulate in injured tissues, such as muscle and brain, through specific chemokine–chemokine receptor systems and tissue-specific antigen recognition by particular T cell receptors (TCRs).^7–9^ For example, Tregs have been shown to accumulate in the brain after ischemic brain injury through CCR6 and CCR8 and to protect against neural cell death by suppressing excessive astrogliosis through the production of a growth factor, amphiregulin (Areg), a low-affinity ligand of the EGF receptor.^7^ The features common to various tissue Tregs are as follows: high expression of genes such as *Il10*, *Il1rl1* (encoding ST2, IL-33 receptor), *Areg*, *Klrg1*, *Ctla4*, *Tigit*, *Gata3*, *Batf*, and *Irf4* and low expression of *Lef1*, *Tcf7*, and *Bcl2* compared with lymphatic tissue Tregs.^10,11^ Analysis of the A384T mutation in the Foxp3 gene has shown that BATF is an important regulator of tissue Tregs.^12^ In addition, the localized microenvironment seems to play pivotal roles in acquiring phenotypes of tissue Tregs.^13^

Tregs have been implicated in the long-term tolerance of autoimmune diseases and allergic diseases and in the suppression of rejection following organ transplantation in humans.^3,14^ While HLA-DR15 confers a markedly increased risk of Goodpasture disease, the HLA-DR1 allele is predominant protective in trans with HLA-DR15. HLA-DR15 presented as an antigen peptide promotes pathogenic Th1/Th17 differentiation, while the HLA-DR1-peptide complex promotes Treg expansion. This study demonstrated a predominant protective effect of HLA in autoimmune disease, which develops self-epitope-specific Tregs that contribute to the protection against or the causation of autoimmunity.^15^ Tregs expressing the Th1-associated chemokine receptor CXCR3 are enriched in the kidneys of patients with antineutrophil cytoplasmic antibody-associated vasculitis and glomerulonephritis (GN) and colocalize with CXCR3^+^ effector T cells.^16^ Th1 and Th17 cells have been demonstrated to be critical for pathogenesis in an experimental mouse model of anti-glomerular basement membrane (GBM)-induced crescentic GN (cGN).^17–19^ Although the protective roles of Tregs in such autoimmune kidney diseases have been well characterized, including the roles of CCR6 and CXCR3 in their recruitment to the kidney,^16,20^ specific subsets of regulatory T cells in the kidney remain to be characterized.

In this study, we reexamined the characteristics of kidney Tregs accumulated in the kidney using an experimental mouse model of cGN induced by anti-GBM antibody. We found that Tregs accumulated in the kidney during the late phase of disease onset (>10 days) and contributed to convalescence by suppressing Th1. Tregs accumulated in the kidney showed particular tissue Treg phenotypes that included high expression of ST2, Areg, KLRG1, and PPARγ. Although RORγt^+^ and T-bet^+^ Tregs were present, the majority of the Tregs in the kidney were GATA3^+^ at the convalescence stage. Although previous studies showed the importance of RORγt and T-bet in Tregs,^21,22^ GATA3 expression levels in Tregs increased during the late stage of the disease in a IL-33-, Areg-, and CCR4-dependent manner. Furthermore, a PPARγ agonist, pioglitazone, enhanced the accumulation of GATA3^+^ Tregs in the kidney and ameliorated cGN. Our data suggest that GATA3 is important for the recruitment of Tregs into the kidney, where they contribute to the maintenance of stably low disease activity in autoantibody-mediated renal tissue destruction.

## Results

### Treg accumulation in the kidney in a murine cGN model

**Fig. 1:**
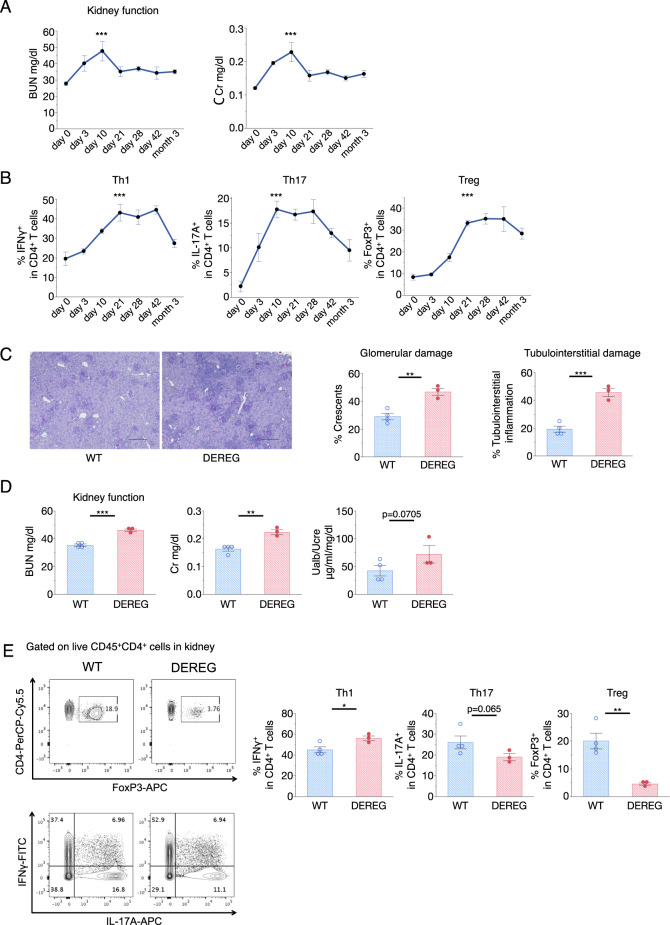
Treg infiltration into the kidney and the effect of Treg depletion during the late phase of cGN. **a** Kidney function was assessed by measuring blood urea nitrogen (BUN) and serum creatinine (Cr) levels at the indicated time points after the administration of an anti-GBM antibody (*n* = 3−5). **b** Changes in proportions of IFNγ^+^, IL-17A^+^, and FoxP3^+^ cells per FVD^−^CD45^+^CD4^+^ cells are presented from isolated kidneys (*n* = 3−5). Symbols represent individual data points for mice in one of two independent experiments, and horizontal lines indicate means ± standard errors of the mean (SEM); ^***^P < 0.001 vs. day 0 (Tukey–Kramer HSD test). **c** Representative photomicrographs (×100 magnification) 26 days after cGN induction following injection of DT on days 21 and 23 in DEREG mice and WT littermates. Sections were stained with periodic acid–Schiff (PAS). Scale bar, 200 μm. Glomerular crescent formation and interstitial inflammation were evaluated in renal pathological assessments (right panels). **d** Kidney function was assessed by measuring serum BUN and Cr levels, and albuminuria was assessed through ratios of urine albumin (UAlb)/urine creatinine (UCre; *n* = 3−4). **e** Representative dot plots from flow cytometry (FCM) analyses gated on FVD^−^CD45^+^CD4^+^ cells that were isolated from kidneys. The proportion of FoxP3^+^, IFNγ^+^, and IL-17A^+^ cells per FVD^−^CD45^+^CD4^+^ cells in kidney tissues (*n* = 3−4). In the right panels, symbols represent individual data points of mice representative of three independent experiments, and horizontal lines indicate the means ± SEM; ^*^P < 0.05, ^**^P < 0.01, and ^***^P < 0.001 (Student’s *t*-test)

To investigate the function of chemokine receptors in Treg-mediated tolerance in cGN, we used a well-characterized T cell-mediated mouse model of anti-GBM antibody-induced nephritis. Although various studies have demonstrated that this model is dependent on Th17 and Th1 cells and is regulated by Tregs,^16,17,19,23^ these studies mostly examined the acute phase, which occurs within the first 10 days of onset. To investigate the role of Tregs during the late phase of cGN disease, we investigated the time course of the severity of the disease and helper T cell infiltration into the kidney following disease induction. As shown in Fig. 1a, disease activity, determined by blood urea nitrogen (BUN) and creatinine (Cr) levels, declined after day 10, and conditions remained calm and stable after 21 days. The levels of pathogenic IL-17A^+^ Th17 cells peaked 10 days after cGN induction, while the IFNγ^+^ Th1 and Treg proportion reached a plateau on day 21 and remained at high levels for at least 3 months (Fig. 1b), which was consistent with a previous report.^18^ Compared with Treg levels in the normal kidney and in secondary lymphoid organs, which are usually ~10%, the Treg levels in the kidney at the late or convalescence phase were 30–35% (Fig. 1b). These data suggest that Tregs tend to accumulate in inflamed kidneys at the late stage of the disease.

To investigate the role of Tregs in the maintenance of the convalescence state during the late phase of cGN, we depleted Tregs using “depletion of regulatory T cell” DEREG mice expressing the diphtheria toxin (DT) receptor under the control of the FoxP3 gene promoter.^24^ Treg depletion at the early phase ameliorated cGN.^15,23^ To verify the roles of Tregs at the late or convalescence stage, we assessed the depletion of Tregs 21 days after nephrotoxic antibody injection (Fig. 1c–e). Treg depletion 21 days after disease onset caused a relapse, which resulted in the rapid induction of tissue damage (Fig. 1c) and aggravation of kidney functions (Fig. 1d). Treg depletion also increased Th1 cells but not Th17 cells (Fig. 1e), indicating that Tregs were necessary not only for the early phase of inflammation but also for maintaining the convalescence stage by suppressing Th1 cells.

### Tregs accumulated in the kidney during the convalescence phase show tissue Treg phenotypes

**Fig. 2:**
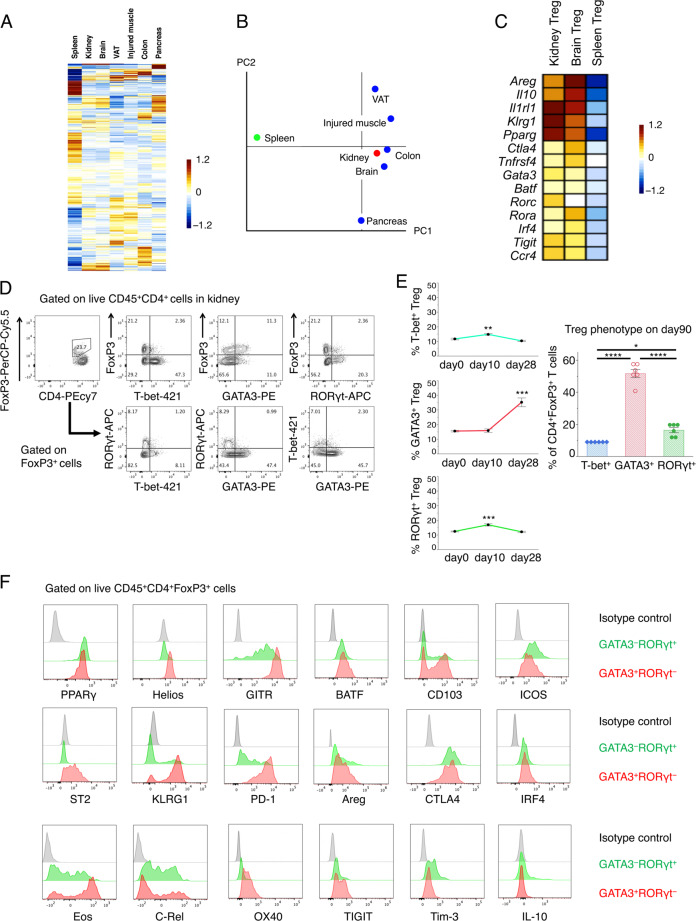
Characterization of kidney Treg subsets and the expression levels of tissue Treg markers Microarray analysis, with the exception of the kidney data, was based on data from our previous paper.^7^ **a**, **b** Heat map and principal component (PC) analysis of the microarray analysis data of Tregs in the kidney, brain, VAT, injured muscle, colon, pancreas, and spleen. Tregs in the kidney were isolated 28 days after cGN induction. **c** Heat map of representative tissue Treg genes obtained by the microarray analysis of Tregs from diseased kidneys, ischemic brains, and spleens. **d** Representative dot plots of FCM analysis gated on FVD^−^CD45^+^CD4^+^ (upper) and FVD^−^CD45^+^CD4^+^FoxP3^+^ (lower) isolated from the kidneys of WT mice 28 days after cGN induction. **e** The proportion of T-bet^+^, GATA3^+^, and RORγt^+^ FoxP3^+^ cells in the kidneys of the WT mice on days 0, 10, 28 (left), and 90 (right) (*n* = 6). **f** Representative histogram expressions of PPARγ, Helios, GITR, BATF, CD103, ICOS, ST2, KLRG1, PD-1, Areg, CTLA4, IRF4, Eos, C-Rel, OX40, TIGIT, Tim-3, and IL-10 in GATA3^−^RORγt^+^ Tregs and GATA3^+^RORγt^−^ Tregs isolated from kidneys 28 days after cGN induction. Symbols represent individual data points of mice representative of two independent experiments, and the horizontal lines indicate the means ± SEM; ^*^P < 0.05, ^**^P < 0.01, ^***^P < 0.001, and ^****^P < 0.0001 (Tukey–Kramer HSD test)

Next, we investigated the phenotypes of Tregs that infiltrated the kidney (kidney Tregs) during the convalescence stage of cGN. Microarray and principal component (PC) analysis revealed that kidney Tregs differ from splenic Tregs but are similar to other tissue Tregs (Fig. 2a, b). Kidney Tregs resemble brain Tregs and express high levels of genes encoding PPARγ, AREG, IL-10, KLRG1, and IL-33 receptor subunit ST2 (Fig. 2c).

Various transcription factors have been reported to drive transcriptomic diversification of particular Treg populations. GATA3 expression is detected in most tissue Tregs,^11^ T-bet (*Tbx21*) is detected in a subset of muscle and colonic Tregs^25,26^ and RORγt (*Rorc*) is detected in distinct subpopulations of colonic and other tissue Tregs.^27–29^ Thus, we measured GATA3^+^, RORγt^+^, and T-bet^+^ factions in Tregs during the course of kidney disease development (Fig. 2d). GATA3, RORγt, and T-bet expression in Tregs were mutually exclusive; that is, there were few double-positive Tregs (Fig. 2d). The fractions of RORγt^+^ or T-bet^+^ Tregs slightly increased from the acute to late phases of the disease, while the GATA3^+^ Treg population was drastically increased by more than 50% at the convalescence stage (Fig. 2e).

GATA3^+^ Tregs expressed higher levels of Helios, glucocorticoid-induced TNFR-related protein (GITR), CD103, ST2, Areg, KLRG1, PD-1, Eos, OX40, and TIGIT than RORγt^+^ Tregs (Fig. 2f). On the other hand, ICOS, cytotoxic T-lymphocyte-associated protein 4 (CTLA4), and IL-10 showed similar expression patterns to those of RORγt^+^ Tregs (Fig. 2f). BATF has been shown to drive the differentiation of tissue Treg precursors^30^ and is necessary for the development of tissue Tregs.^12^ As shown in Fig. 2f, both GATA3^+^ Tregs and RORγt^+^ Tregs were BATF-positive; however, the BATF levels in GATA3^+^ Tregs were slightly higher than those in RORγt^+^ Tregs (Fig. 2f). BATF mRNA levels were low but detectable in splenic Tregs, and these levels were drastically increased in kidney Tregs (Fig. 2c), suggesting that BATF is expressed in LNs at low levels but were dramatically increased after infiltration into the kidney.

**Fig. 3:**
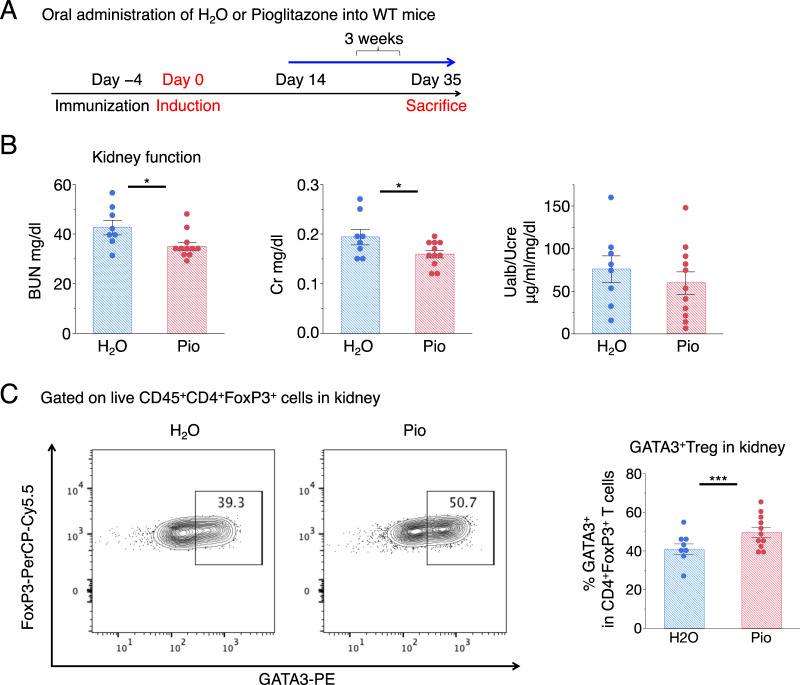
Effect of PPARγ agonists on disease severity and GATA3^+^ Treg accumulation. **a** Schematic procedure for the pioglitazone treatment regimen. Oral administration of H_2_O or pioglitazone via a feeding needle at doses of 30 μg/g body weight per day every day for 3 weeks. **b** Kidney function was assessed by measuring serum BUN and creatinine levels and determining ratios of urine albumin (UAlb)/urine creatinine (UCre) (*n* = 8–11). **c** Representative dot plots of FCM analysis gated on FVD^−^CD45^+^CD4^+^FoxP3^+^ cells isolated from the kidneys of WT mice 35 days after being treated with H_2_O or pioglitazone. The proportion of GATA3^+^ per FVD^−^CD45^+^CD4^+^FoxP3^+^ cells in the kidney. Symbols represent individual data points of the mice, and horizontal lines indicate the means ± SEM; ^*^P < 0.05 and ^***^P < 0.001 (Student’s *t*-test)

PPARγ agonists have been reported to increase the accumulation of adipose-tissue Tregs and improve insulin sensitivity,^31^ and PPARγ agonists have a protective effect against various types of kidney injuries.^32,33^ Since kidney Tregs express a high level of PPARγ, similar to that of visceral adipose tissue (VAT) Tregs (Fig. 2c), we examined the effect of the PPARγ agonist pioglitazone on Treg accumulation in the kidney (Fig. 3a). As reported, pioglitazone reduced disease activity (Fig. 3b). We noticed that pioglitazone did not have much of an effect on Foxp3^+^ Treg levels in the kidney but significantly increased the GATA3^+^ fraction in the Treg population (Fig. 3c). Thus, we focused on the role of GATA3 in kidney Tregs.

### GATA3 in Tregs is important for convalescence after attenuated kidney inflammation

To demonstrate the importance of GATA3^+^ Tregs in the kidney at the convalescence stage, we used DEREG mice and tamoxifen-inducible *Gata3*-deficient (*Gata3*^*f/f*^-CreER^T2^) mice.^34^ First, we injected various amounts of DT into the DEREG mice and found that GATA3^+^ Tregs were more profoundly affected by DT treatment than were total kidney Tregs (Supplementary Fig. 1). For example, 0.5 μg of injected DT reduced Foxp3^+^ Tregs to ~70–80% of the control, while GATA3^+^ Tregs were reduced to 40–50% of the control (Supplementary Fig. 1B). Under this condition, disease severity, as measured by BUN, Cr, and UAlb levels, was increased (Supplementary Fig. 1A). These disease activity indicators correlated well with the GATA3^+^ Treg population but not the total Treg population in the kidney (Supplementary Fig. 1C). Second, the deletion of GATA3 by tamoxifen during the convalescence stage of the *Gata3*^*f/f*^-CreER^T2^ mice excerbated the disease and almost completely diminished GATA3^+^ Tregs without affecting total Tregs in the kidney (Supplementary Fig. 2). These data suggest that GATA3^+^ Tregs, but not the total number of Tregs, are important for attenuating disease severity.

### Development of a transfer model that shows the role of GATA3 in Treg functions in the kidney

**Fig. 4:**
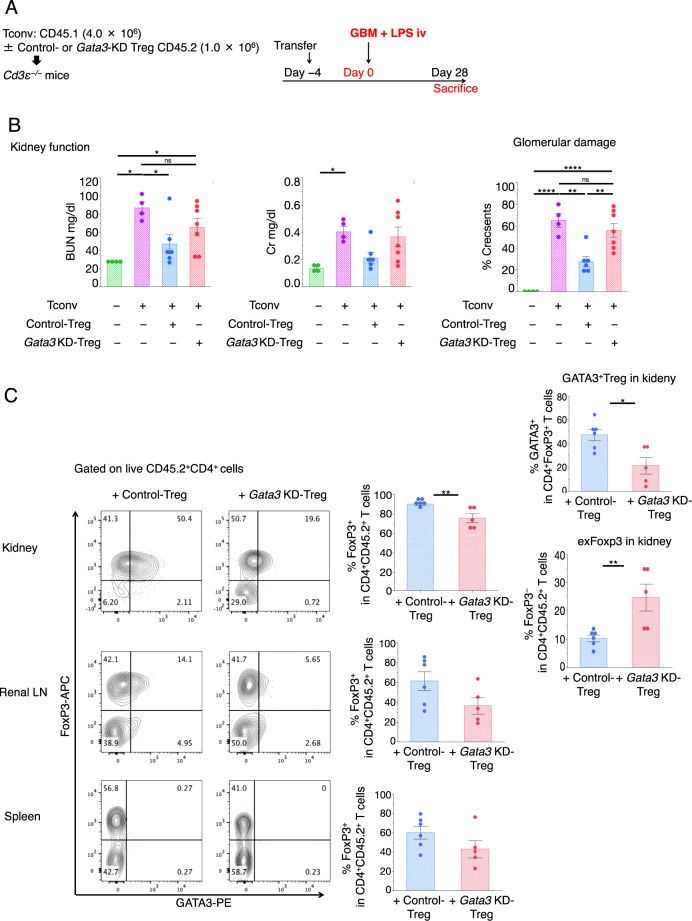
cGN induction by T cell transfer and the effect of GATA3 in the Tregs. **a** Schematic procedure for induction of cGN in *Cd3*ε^−/−^ mice by adoptive transfer of CD45.1^+^CD4^+^CD25^−^ Tconvs (4.0 × 10^6^) with or without CD45.2^+^CD4^+^FoxP3-hCD2^+^ Tregs (1.0 × 10^6^) from spleens and lymph nodes from intact mice transduced with CRISPR–Cas9 (control gRNA or *Gata3* gRNA). **b** Kidney function 28 days after cGN induction was assessed by measuring serum BUN and creatinine levels, and glomerular crescent formation was evaluated according to renal pathological findings. (*n* = 4–7). **c** Representative dot plots of FCM analysis gated on the FVD^−^CD45.2^+^CD45.1^−^CD4^+^ cells in the kidney (upper), renal lymph node (middle), and spleen (lower). The proportion of FoxP3^+^ cells per FVD^−^CD45.2^+^CD45.1^−^CD4^+^ cells in the kidney (upper) and the proportion of GATA3^+^ cells per FVD^−^CD45.2^+^CD45.1^−^CD4^+^FoxP3^+^ cells (right upper) and FoxP3^−^ cells per FVD^−^CD45.2^+^CD45.1^−^CD4^+^ cells in the kidney (right lower) (*n* = 5–6). The proportions of FoxP3^+^ cells per FVD^−^CD45.2^+^CD45.1^−^CD4^+^ cells in the renal lymph node (middle) and spleen (lower) are shown. Symbols represent individual data points of mice representative of two independent experiments, and horizontal lines indicate the means ± SEM; ^*^P < 0.05, ^**^P < 0.01, ^***^P < 0.001, and ^****^P < 0.0001 (Student’s *t*-test and Tukey–Kramer HSD test)

To investigate the role of GATA3 specifically in kidney Treg functions, we developed a CD4^+^ T cell reconstitution system to induce cGN. Conventional CD45.1^+^CD25^−^CD4^+^ T cells (Tconvs) were transferred together with CD45.2^+^CD25^+^CD4^+^ Tregs isolated from the spleen and LNs at a 4:1 ratio into *Cd3ε*^−/−^ mice 4 days before anti-GBM antibody administration. The indicators were measured 28 days after disease induction (Fig. 4a). The *Cd3ε*^−/−^ mice did not show distinct disease phenotypes; however, the transfer of conventional T cells alone resulted in severe renal dysfunction, and the cotransfer of WT Tregs reduced the severity of cGN, as indicated by BUN and Cr levels and crescent formation (Fig. 4b).

This system was very useful for evaluating the function of genes of interest specifically in Tregs. The *Gata3* gene in Tregs was deleted using the CRISPR/Cas9 system as shown previously.^7^ Compared with control Tregs, *Gata3*-knockdown (KD) Tregs did not suppress disease activity very efficiently (Fig. 4b). To estimate the deletion efficiency by the CRISPR/Cas9 system, we compared GATA3 expression levels in kidney Tregs after cGN induction since GATA3 was not expressed in naïve Tregs (Supplementary Fig. 3A). As shown in Supplementary Fig. 3B, the GATA3 deletion efficiency was ~50–60% in the transferred Tregs, as indicated by GATA3 protein levels and a mean fluorescence intensity (MFI) assay of GATA3^+^ Treg fractions.

As shown in Fig. 4c, GATA3 knockdown did not drastically reduce the Foxp3 levels in the CD45.2-transfected Tregs and slightly reduced the level of Foxp3^+^ Tregs in the spleen and renal LNs; however, the reduction in Foxp3 in the spleen and LNs was not statistically significant. Similarly, the reduction in Foxp3^+^ Tregs in the kidney was only <20%, while the reduction in GATA3 in the kidney Tregs was more than 70% (Fig. 4c and Supplementary Fig. 3B). The conversion rate of transferred WT Tregs to Foxp3^−^ T cells (exTregs) was ~10%, while that of GATA3-KD Tregs was 20–30% (Fig. 4c), suggesting that GATA3 knockdown facilitated the conversion of Tregs into exTregs. On the other hand, the conversion of CD45.1^+^ Tconvs to Foxp3^+^ Tregs was <3% (Supplementary Fig. 3C). Since GATA3-KD Tregs failed to suppress cGN severity (Fig. 4b), these data suggest that GATA3 in Tregs plays important role in the suppression of renal inflammation.

T-bet^+^ Tregs and RORγt^+^ Tregs have been shown to suppress cGN disease in the acute phase.^21,22^ To examine the significance of T-bet and RORγt at the convalescence phase, we performed transfer experiments using Tregs from Tbx21- and *Rorc*-deficient mice. We transferred WT, *Tbx21*^−/−^ or *Rorc*^−/−^ Tregs together with Tconvs to CD3-deficient mice. As shown in Supplementary Fig. 4, the *Rorc*^−/−^ Tregs suppressed cGN similar to WT Tregs, while the *Tbx21*^−/−^ Tregs failed to suppress the disease. These data indicate that, in addition to GATA3, T-bet is important in the suppressive function of kidney Tregs during the convalescence phase, while RORγt is more important during the early phase.

### ST2 and Areg are essential for kidney Treg accumulation and function

Since ST2 and Areg are highly expressed in GATA3^+^ Tregs, we investigated the role of IL-33/ST2 and Areg in kidney Treg development and function. There are few reports showing investigation into the roles of IL-33/ST2 and Areg in GN. Although the EGF receptor in glomerular epithelial cells (podocytes) has been shown to promote cGN,^35^ little is known about the role of Areg in kidney Tregs.

**Fig. 5:**
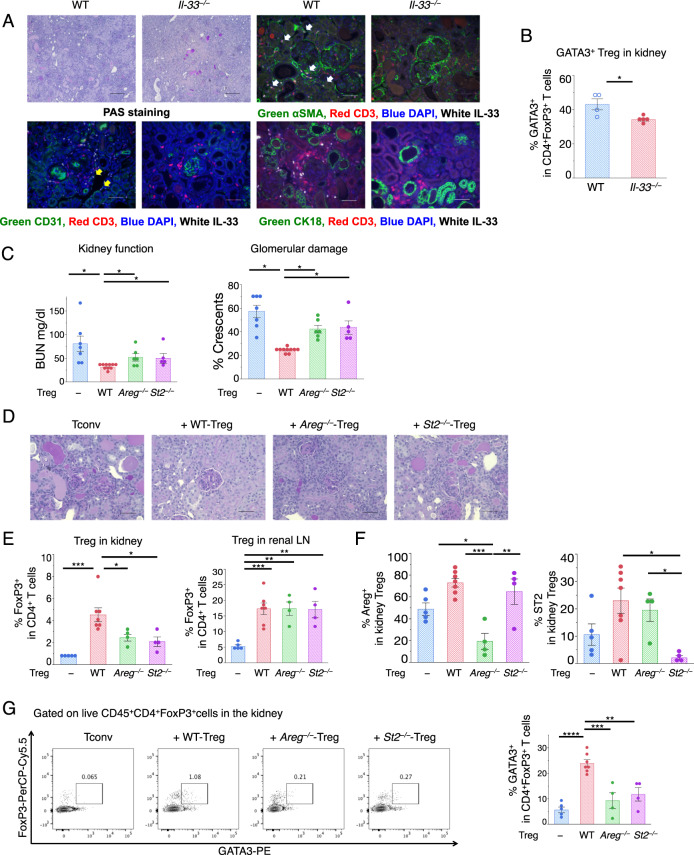
The effect of IL-33 and Areg on kidney damage and Treg populations. **a** Analysis of *Il-33*^−/−^ mice sacrificed 56 days after cGN induction. PAS staining was performed to assess crescentic glomeruli and interstitial inflammation. Representative light microscopic photomicrographs (×100 magnification) are shown in the two uppermost left panels. Scale bar, 200 μm. Representative immunofluorescent photomicrographs (two uppermost right panels and lower panels) were taken at ×400 magnification using indirect immunofluorescence of crescentic glomeruli stained as follows: green, αSMA (upper right), CD31 (lower left), and CK18 (lower right); red, CD3; and blue, DAPI. Scale bar, 50 μm. **b** The proportion of GATA3^+^ cells per FVD^−^CD45^+^CD4^+^FoxP3^+^ cells in the kidney on day 56 (*n* = 5). **c** CD4^+^CD25^−^ Tconvs with or without CD4^+^CD25^+^ Tregs were adoptively transferred into *Cd3ε*^−/−^ mice (*n* = 5–10). Kidney function on day 28 after cGN induction was assessed by measuring serum BUN, and glomerular crescent formation was evaluated according to renal pathological findings. **d** Representative photomicrographs were taken at ×400 magnification using a light microscope and depict PAS-stained crescentic glomeruli and interstitial inflammation obtained from *Cd3ε*^−/−^ mice after adoptive transfer. Scale bar, 50 μm. **e** The proportion of FVD^−^CD45^+^CD4^+^FoxP3^+^ cells in the kidney (left) and renal lymph nodes (LNs) from *Cd3ε*^−/−^ mice after adoptive transfer of T cells and 28 days after cGN induction (*n* = 4–7). **f** The proportion of Areg^+^ cells and ST2^+^ cells per FVD^−^CD45^+^CD4^+^FoxP3^+^ cells in the kidney. **g** Representative dot plots of FCM analysis gated on FVD^−^CD45^+^CD4^+^FoxP3^+^ cells isolated from the kidneys of *Cd3ε*^−/−^ mice 28 days after the adoptive transfer. The proportion of GATA3^+^ cells per FVD^−^CD45^+^CD4^+^FoxP3^+^ cells in the kidney is shown in the right panel. Symbols represent individual data points from mice that are representative of two independent experiments, and horizontal lines indicate the means ± SEM; ^*^P < 0.05, ^**^P < 0.01, ^***^P < 0.001, and ^****^P < 0.0001 (Student’s *t-*test and Tukey–Kramer HSD test)

First, we compared cGN disease severity between IL-33-deficient and WT mice. The results showed that the BUN and Cr levels were not significantly different, although albuminuria and tubulointerstitial damage were increased in the *Il33*^−/−^ mice during the late phase (Fig. 5a, PAS staining and Supplementary Fig. 5A). Although the number of Th1/Th17/Tregs in the kidney was not significantly changed (data not shown), the fraction of GATA3^+^ Tregs was decreased in the *Il33*^−/−^ mice (Fig. 5b and Supplementary Fig. 5b). Immunohistochemical analysis revealed that IL-33 was predominantly expressed in αSMA-positive fibroblasts and expressed at low levels in CD31-positive endothelial cells in the injured kidney (Fig. 5a).

To examine the roles of ST2 and Areg in kidney Tregs, we used the CD4^+^ T cell reconstitution system described in Fig. 4. Conventional T cells (Tconvs) and WT or gene-deficient Tregs were transferred into *Cd3ε*^−/−^ mice 4 days before anti-GBM antibody administration, and key indicators were examined 28 days after disease induction (see Fig. 4a). The WT Tregs showed suppressed BUN and crescent formation, while the *St2*^−/−^ and *Areg*^−/−^ Tregs did not show suppressed BUN levels or crescent formation (Fig. 5c, d). Treg accumulation in the kidney was reduced to ~50% when *St2* or *Areg* in the Tregs was deleted, while the expression of these proteins in associated LNs was not affected (Fig. 5e). The expression levels of Areg and ST2 were severely reduced in *Areg*^−/−^ Treg-transferred kidneys and *St2*^−/−^ Treg-transferred kidneys, respectively (Fig. 5f and Supplementary Fig. 6A). A more detailed analysis revealed that GATA3 expression was reduced in the *St2*^−/−^ and *Areg*^−/−^ Tregs in the kidney, while RORγt and T-bet expression were not drastically changed (Fig. 5g and Supplementary Fig. 6B). Thus, IL-33 and Areg are required for the expansion and/or accumulation of GATA3^+^ Tregs. We observed ~5% conversion of Tconvs into Foxp3^+^ T cells when Tconvs were transferred into mice (Fig. 5e, f). This conversion rate may be the result of the generation of induced Tregs or transient expression of Foxp3 and/or expansion of contaminated Foxp3^+^CD25^low^ Tregs in severely inflamed conditions.

When cGN was induced in *Areg*^−/−^ mice, Foxp3 and GATA3 levels were severely affected compared with those in WT mice (Supplementary Fig. 7A). In addition, *Areg*^−/−^ Tregs did not proliferate efficiently in response to IL-2 and TCR stimulation compared to the WT Tregs (Supplementary Fig. 7B). These data are consistent with a previous report showing that Areg supports Treg proliferation and survival^36^ and strongly support our proposal that Areg plays an important role in GATA3^+^ kidney Tregs.

### CCR4 is an important chemokine receptor that facilitates the accumulation of Tregs in the kidney during the late phase

**Fig. 6:**
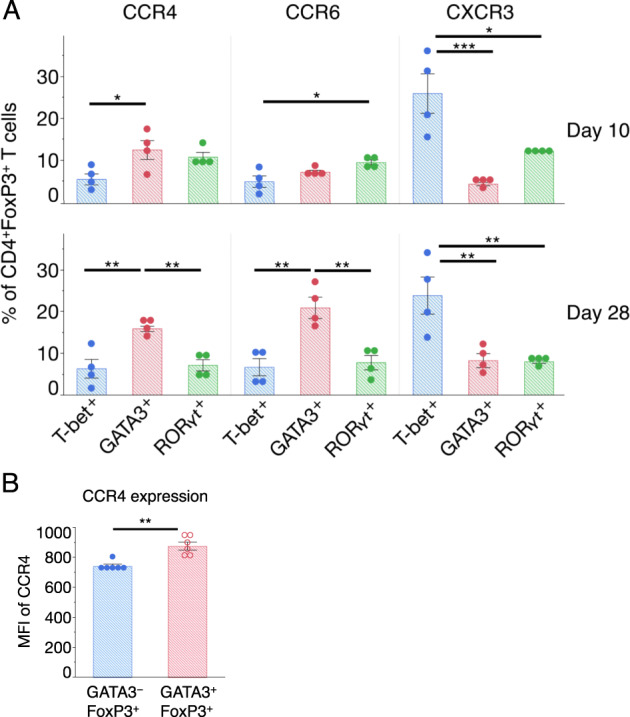
Expression of representative chemokine receptors on kidney Treg subsets. **a** The proportion of CCR4, CCR6, and CXCR3^+^ cells to T-bet^+^, GATA3^+^, and RORγt^+^ cells per FVD^−^CD45^+^CD4^+^FoxP3^+^ cells in the kidney on 10 and 28 days after cGN induction. Representative FCM plots are shown in Supplementary Fig. 4. **b** Quantification of the mean fluorescence intensities (MFIs) of CCR4 expression on the GATA3^−^FoxP3^+^ and GATA3^+^FoxP3^−^ cells in the kidney on day 28. Symbols represent individual data points of mice representative of two independent experiments, and horizontal lines indicate the means ± SEM; ^*^P < 0.05, ^**^P < 0.01, and ^***^P < 0.001 (Student’s *t-*test and Tukey–Kramer HSD test)

Next, we examined chemokine receptors that are highly expressed in kidney Tregs. As shown in Fig. 6a and Supplementary Fig. 8, CCR4 and CCR6 were expressed on both GATA3^+^ and RORγt^+^ Tregs, while CXCR3 was predominantly expressed in T-bet^+^ Tregs at the acute phase (day 10). However, the CCR4 and CCR6 expression levels were increased in GATA3^+^ Tregs but not in the RORγt^+^ Tregs during the late phase (day 28). The expression levels of CCR4 were higher in GATA3^+^ Tregs than in GATA3^−^ Tregs (Fig. 6b), suggesting that CCR4 expression is regulated by GATA3.

**Fig. 7:**
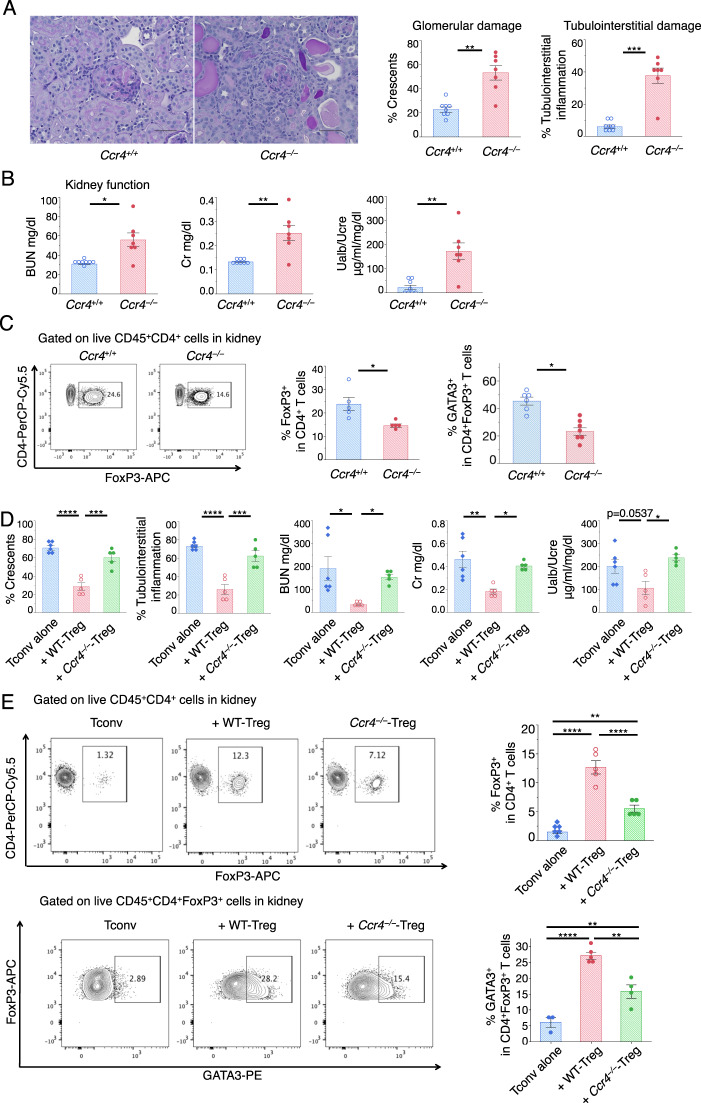
Role of CCR4 in kidney Tregs. **a** Analysis of *Ccr4*^−/−^ mice sacrificed on day 10. Representative photomicrographs were taken at ×400 magnification using a light microscope and depict PAS-stained crescentic glomeruli and interstitial inflammation. Scale bar, 50 μm. Glomerular crescent formation and interstitial inflammation were evaluated according to renal pathological findings (*n* = 7–8). **b** Kidney function was assessed by measuring serum BUN and creatinine levels and the ratios of urine albumin (UAlb)/urine creatinine (UCre) (*n* = 7–8). **c** Representative dot plots from FCM analyses gated on FVD^−^CD45^+^CD4^+^ cells that were isolated from kidneys. The proportion of FoxP3^+^ cells (left) per FVD^−^CD45^+^CD4^+^ cells (*n* = 5) and GATA3^+^ cells (right) per FVD^−^CD45^+^CD4^+^FoxP3^+^ cells in the kidney (*n* = 6–7). Symbols represent individual data points of mice representative of three independent experiments, and horizontal lines indicate the means ± SEM; ^*^P < 0.05, ^**^P < 0.01, and ^***^P < 0.001 (Student’s *t-*test). **d** Renal pathological observations revealed elevated glomerular crescent formation and interstitial inflammation. Kidney function 28 days after cGN induction was assessed by measuring serum BUN and creatinine levels and the ratios of urine albumin (UAlb)/urine creatinine (UCre) from *Cd3ε*^−/−^ mice adoptively transferred with CD4^+^CD25^+^ (Tregs: 1.0 × 10^6^) with or without CD4^+^CD25^−^ (Tconvs: 4.0 × 10^6^) cells from the spleens and lymph nodes of the WT or *Ccr4*^−/−^ mice (*n* = 5–6). **e** Representative dot plots of FCM analyses gated on FVD^−^CD45^+^CD4^+^ cells and FVD^−^CD45^+^CD4^+^FoxP3^+^ cells from kidneys. The proportion of FoxP3^+^ cells per FVD^−^CD45^+^CD4^+^ cells and GATA3^+^ cells per FVD^−^CD45^+^CD4^+^FoxP3^+^ cells in the kidney (*n* = 3–6). In the right panel, symbols represent individual data points of mice representative of three independent experiments, and horizontal lines indicate the means ± SEM; ^*^P < 0.05, ^**^P < 0.01, ^***^P < 0.001, and ^****^P < 0.0001 (Tukey–Kramer HSD test)

Although the roles of CCR6 and CXCR3 in the recruitment of Tregs into the kidney have already been established in similar antibody-mediated nephritis models,^16,20^ CCR4 in Tregs during the late phase has not been investigated. We, therefore, focused on CCR4 in Tregs. We compared cGN symptoms between WT and *Ccr4*^−/−^ mice. Histological analysis revealed that *Ccr4*^−/−^ mice developed more severe glomerular and tubulointerstitial damage than WT mice (Fig. 7a), and serum BUN and Cr levels and urine albumin levels were much higher in *Ccr4*^−/−^ mice than they were in WT mice (Fig. 7b). The proportion of FoxP3^+^ Tregs, as well as that of GATA3^+^ Tregs, was lower in the kidneys of the *Ccr4*^−/−^ mice than it was in the kidneys of the WT mice (Fig. 7c).

To define the role of CCR4 in Tregs, we used a reconstituted cGN model system (Fig. 7d, e). CCR4-deficient Tregs failed to control cGN symptoms, including crescent formation, tubulointerstitial damage, BUN and Cr levels, and albuminuria (Fig. 7d). CCR4 deficiency decreased the accumulation of total Tregs as well as GATA3^+^ Tregs in the kidney (Fig. 7e). Therefore, the presence of CCR4 on Tregs is crucial not only for cGN regulation and Treg migration into the kidney but also for the selective accumulation of GATA3^+^ Tregs in the kidney.

## Discussion

cGN is among the most severe pathological kidney diseases and results in end-stage renal failure, which is associated with high morbidity, mortality, and medical expenses. The anti-GBM antibody-mediated murine cGN model has been widely used to understand the role of T cell-immune systems in nephritis. However, most research has focused on the fulminant stage (within 10 days of disease onset), and only a few studies have analyzed the late or convalescence stage (3 weeks after onset). In this study, we developed a novel method to study genes in Tregs by using the transfer of Tconvs together with Tregs to T-cell-deficient mice. We demonstrated an important role for kidney tissue-accumulated GATA3^+^ Tregs in the maintenance of stable disease during the late stage, where the number of Tregs and Th1 cells seems to equilibrate.

Previous studies highlighted the important role of RORγt^+^Foxp3^+^ double-positive (bi)Tregs, which carry the trafficking receptor CCR6, in the suppression of pathogenic Th17 cells in cGN models.^22,37^ RORγt^+^ Tregs were generally thought to suppress Th17 cells specifically. Interestingly, RORγt^+^Foxp3^+^ biTregs have been shown to produce both anti-inflammatory (IL-10, IL-35) and proinflammatory (IL-17) cytokines, but the transfer of biTregs suppressed the development of nephritis to an extent similar to that of the transferred conventional Tregs.^22^ However, RORγt in Tregs may play a pathogenic role in cGN since the deletion of *Rorc* in Tregs ameliorated cGN symptoms.^22^ T-bet^+^ Tregs are also present in several organs and specifically suppress Th1 cells,^38^ and T-bet has been shown to promote CXCR3 expression.^25^ It has also been reported that T-bet expression increases during the course of nephritis, and the deletion of T-bet in Tregs results in aggravated nephritis with enhanced renal and systemic Th1 responses.^21^ Our data suggest that *Rorc*^−/−^ Tregs suppressed cGN symptoms at a late stage, similar to WT Tregs, while *Tbx21*^−/−^ Tregs failed to suppress symptoms (Supplementary Fig. 4). These data indicate that, in addition to GATA3, T-bet is important for the suppressive function of kidney Tregs during the convalescence phase, while RORγt is more important for disease suppression during the early phase. However, further study is necessary to elucidate the roles of these transcription factors in the function of kidney Tregs.

We focused on GATA3^+^ Tregs in the kidney since the expression of GATA3 in Tregs increased during the convalescence stage and because the expression levels of ST2, Areg, and CCR4 were high. GATA3 has been shown to be important for stable Foxp3 expression and the suppression of various types of inflammation, including not only Th2 but also Th17 types.^29,39^ We found that GATA3 expression is highly correlated with the IL-33 receptor, ST2, Areg, and chemokine receptor CCR4. GATA3 in Tregs is essential for maintaining stable disease conditions during the convalescence stage. All factors that we examined in this study, ST2, Areg and CCR4, affected GATA3^+^ Tregs in the kidney. One of the important functions of GATA3 in kidney Tregs may be inducing the expression of these factors and, in return, these factors may facilitate GATA3^+^ Treg expansion and accumulation in the kidney. Hence, we propose a virtuous circle of kidney Tregs.

A previous study showed that the forced expression of ST2 in human Tregs confers the ability of IL-33 to stimulate Areg expression in Tregs, which promotes the acquisition of an M2-like macrophage phenotype.^40^ Therefore, IL-33 seems to be involved not only in the expansion but also in the repair of tissue Tregs. However, a few studies have investigated the effect of IL-33 on GN. One study demonstrated the therapeutic potential of a hybrid cytokine with active domains of IL-2 and IL-33 that induced the remission of lupus-mediated GN in NZM2328 and MRL/lpr mice.^41^ The remission was accompanied by the persistent elevation of Tregs in renal lymph nodes. In this study, we demonstrated the potential therapeutic ability of IL-33 on antibody-induced cGN by upregulating GATA3^+^ kidney Tregs. However, as shown in Fig. 5, there are some discrepancies between the untreated *Il33*^−/−^ mice and the mice into which ST2-deficient Tregs were transferred. Treg transfer experiments suggested that ST2^+^ Tregs suppress BUN and Cr levels and prevented proteinuria. However, IL-33 in mice is more intricately involved in proteinuria development than in changes to BUN and Cr levels. This discrepancy may be due to the expression of ST2 in various types of cells; IL-33 may play not only protective but also pathogenic roles, depending on the cell type.^42^ ST2 is expressed not only in Tregs but also in pathogenic Th2 cells, type 2 innate lymphoid cells, and mast cells; therefore, IL-33 has been discussed in terms of both its beneficial and harmful effects.^43,44^ Thus, it is important to target IL-33 signals specifically in Tregs for therapeutics.

The mechanism by which kidney Tregs attenuates renal injury remains to be clarified. IL-10 is an important candidate, since various reports have demonstrated that IL-10 from Tregs plays essential roles in attenuating autoimmune-mediated cGN.^45–48^ TGF-β has also been shown to be involved in the suppression of inflammation and tissue remodeling^49^ and to mediate renal hypertrophy, glomerulosclerosis, and tubulointerstitial fibrosis.^50^ Areg has been shown to be involved not only in type 2 immunity but also in tissue tolerance.^51^ It has also been reported that muscle Tregs promote muscle repair by producing Areg.^8^ Muscle Tregs also suppress excessive activation of tissue macrophages,^52^ and brain Tregs have been shown to suppress excess activation of astrocytes in the injured brain.^7^ Since Areg is highly expressed in kidney Tregs, it is a strong candidate for the resolution of inflammation and tissue repair factors produced from kidney Tregs. Indeed, we observed that Areg-deficient Tregs failed to attenuate kidney injury (Fig. 5). Notably, Areg can be an autocrine factor for tissue Tregs because Tregs have been shown to express the EGF receptor and Areg enhances Treg functions.^36^ We also confirmed the important roles of Areg in GATA3^+^ Treg development of autocrine-promoting proliferation (Supplementary Fig. 7). However, since Areg is produced not only from Tregs but also from various types of cells,^53^ we cannot conclude that Areg is an essential factor for kidney Tregs.

We discovered an important function of CCR4 in kidney Tregs. Recent single-cell transcriptomics has revealed the trajectories of tissue adaptation in mice and humans; for example, the priming of Tregs to nonlymphoid tissues occurs in the LNs.^11,13^ Previous studies demonstrated the importance of CCR6 and CXCR3 in Tregs for the attenuation of cGN symptoms at the fulminant phase.^16,20,21^ In addition to the chemokine receptors demonstrated by these studies, we proposed that CCR4 signals in Tregs are important for the migration of Tregs from renal LNs into the kidney, especially during the late stage. In addition, we found that the infiltration of GATA3^+^ Tregs was impaired in the *Ccr4*^−/−^ mice, suggesting that CCR4 signals might promote the selective recruitment of GATA3^+^ Tregs to the kidney or the expression of GATA3^+^ in Tregs.

In conclusion, we have demonstrated that Tregs accumulated in the kidney during the convalescence or late phase, which had acquired tissue Treg phenotypes and may be involved in the stability of the disease, and GATA3, ST2, Areg, and CCR4 are thought to be important effector molecules for kidney Tregs. The clinical application of Tregs, including chimeric antigen receptor (CAR)-Tregs, is underway.^54,55^ Our method of knocking out genes in Tregs using the CRISPR/Cas9 system may also facilitate the use of Tregs for the therapy of autoimmune diseases.

## Materials and methods

### Mice

C57BL/6J mice were purchased from Tokyo Laboratory Animals Science Co., Ltd.*, Foxp3-DTR/EGFP* (DEREG), *Foxp3-hCD2*, *Tbx21*^−/−^*, Rorc*^−/−^*, Areg*^−/−^*, Il-33*^−/−^*, St2*^−/−^, and *Cd3ε*^−/−^ mice were described previously.^7,24,56,57^ The *Ccr4*^−/−^ mice were obtained as previously described.^7,58^ Tamoxifen-inducible *Gata3*-deficient (*Gata3*^*f/f*^-CreER^T2^) mice have been described previously.^34^ All mice had a C57BL/6 genetic background. Male mice, aged 8–12 weeks and weighing 20–30 g, were used under cohousing conditions in specific pathogen-free facilities.

Animal experiments were performed in strict accordance with the recommendations in the Guidelines for Proper Conduct of Animal Experiments of the Science Council of Japan. All experiments were approved by the Animal Research Committee and Ethics Committee of Keio University.

### Induction of the experimental cGN model

Rabbit anti-murine GBM antibody was prepared through in-house immunization of rabbits with purified mouse GBM.^59^ cGN was induced by a standard method.^60^ In some experiments, we preimmunized mice with normal rabbit globulin (0.25 mg) using complete Freund’s adjuvant and *Mycobacterium tuberculosis* (H37Rv) at 0.5 mg/mouse (Difco Laboratories) to facilitate the disease. Four days after preimmunization, rabbit anti-mouse GBM IgG (3 mg/mouse) with 75 ng of lipopolysaccharide (Sigma-Aldrich *Escherichia coli* O26:B6 L8274) was injected intravenously.

To measure renal function, serum and urinary creatinine (Cr), serum BUN, and urine albumin/creatinine were measured using standard laboratory methods by Oriental Yeast Co., Ltd.

### Histologic assessment and flow cytometry (FCM) analysis

The renal cortex was fixed in paraformaldehyde overnight, and 4-μm tissue sections were cut and stained with periodic acid–Schiff (PAS). Assessment of renal injury was carried out according to the methods described by Ooi et al.^61^ In brief, a glomerulus was considered to exhibit crescent formation when two or more layers of cells were observed in Bowman’s space. A minimum of 100 glomeruli was assessed to determine the crescent score in a blinded manner. To evaluate tubulointerstitial inflammation, 15 sites on the cortical area were randomly selected, and the average percentage of leukocytic infiltration, tubular dilation, tubular atrophy, sloughing of tubular epithelial cells and cast formation were determined at ×200 magnification using a BZ-X700 fluorescence microscope (Keyence). For immunohistochemical analysis, paraffin-embedded mouse kidney tissue was used after heat-mediated antigen retrieval with 10 mM citrate buffer (pH 6.0) for 15 min at 100 °C.

For FCM analysis, kidney cells were obtained from the mice with saline perfusion and then digested with 5 mg ml^−1^ collagenase D and 100 mg ml^−1^ DNase I (both from Roche Diagnostics) in RPMI 1640 supplemented with 10% FBS, nonessential amino acids, penicillin/streptomycin, and 2-mercaptoethanol (55 µM: Invitrogen) for 30 min at 37 °C and finally filtered at 70 μm. For chemokine staining, kidney tissue was cut into small pieces (<1 mm) and then directly filtered at 70 μm in RPMI 1640 with 5% FBS. Thereafter, the CD45^+^ leukocyte was isolated using an autoMACS Pro Separator with anti-mouse CD45 microbeads (30F11.1) antibody (Miltenyi Biotec) by positive selection (Possel). Before using the microbeads, staining was performed in the presence of the Fc-blocking antibody (2.4G2). For intracellular staining (IFNγ, IL-17A and Areg), cells were incubated with 10% FBS/RPMI including PMA (50 ng/ml; Sigma) and ionomycin (500 ng/ml; Sigma) in the presence of brefeldin A (eBioscience) for 3–4 h, followed by surface and intracellular staining as described.^62^ FCM data were acquired with a FACSCanto II (BD Biosciences) and analyzed with FlowJo 9.9.3 software (FlowJo).

All antibodies and reagents are described in Supplementary Table 1.

### In vivo depletion of Tregs and adoptive transfer

DEREG mice have been described previously.^7,15,23^ Administration of DT was performed as described.^61^ DEREG mice and WT littermates were injected intraperitoneally with 1 µg of DT (Merck Millipore) on day 21 and day 23 and analyzed on day 26. Supplementary Fig. 1 shows that DEREG mice were injected intraperitoneally with PBS, 0.5 µg, 1 µg or 2 µg (1 µg on consecutive 2 days) of DT on day 10 and analyzed on day 21.

For transfer experiments, naïve Tregs were isolated from a population of CD4^+^ T cells obtained from the spleen of naïve WT, FoxP3-KI, *Areg*^−/−^*, St2*^−/−^ and *Ccr4*^−/−^ mice using an autoMACS Pro Separator with a CD4 isolation kit (Miltenyi Biotec) by negative selection (Depletes) and then sorted using phycoerythrin (PE)-conjugated anti-CD25 or anti-hCD2 and PE-Cy7 or PerCP-Cy5.5 anti-CD4 (RM4–5) with a Sony SH800 Cell Sorter (Sony Biotechnology) (purity > 97%) as described previously.^56^ The indicated numbers of CD4^+^CD25^+^, CD4^+^CD25^−^, CD4^+^FoxP3-hCD2^+^, or CD4^+^ FoxP3-hCD2^−^ cells were injected intravenously into *Cd3ε*^−/−^ mice 4 days before anti-GBM injection. For GATA3-KD experiments, we used CD45.1^+^CD25^−^CD4^+^ T cells for the Tconvs and CD45.2^+^CD25^+^CD4^+^FoxP3-hCD2^+^ cells for the Tregs. The Tconvs (4.0 × 10^6^ cells) and Tregs (1 × 10^6^ cells) were transferred into *Cd3ε*^−/−^ mice 4 days before anti-GBM antibody administration and analyzed on day 28.

### Deletion of the Gata3 gene by the CRISPR–Cas9 system

To delete *Gata3* in Tregs, we introduced the guideRNA/Cas9 complex into Tregs according to a previously described procedure.^7^ CRISPR RNA (crRNA) and *trans*-activating crRNA (tracrRNA) were purchased from Integrated DNA Technologies, and the crRNA–tracrRNA duplex was prepared according to the manufacturer’s instructions. Guide sequences for *Gata3* were as follows: crRNA 1: GACUUACAUCCGAACCCGGUGUUUUAGAGCUAUGCU, crRNA 2: AACCACGUCCCGUCCUACUAGUUUUAGAGCUAUGCU, and crRNA 3: ACGUCCACCUCUUCCGUCAGGUUUUAGAGCUAUGCU. The guide sequence for the negative control was as follows: CAUAUUGCGCGUAUAGUCGC. To generate the RNP complex of Cas9–RNP, the crRNA–tracrRNA duplex (180 pmol) was incubated with 10 ng of TrueCut Cas9 Protein v2 (Thermo Fisher Scientific) at room temperature for 15 min. For nucleofection, Tregs (5.0 × 10^6^ per well) were isolated from the spleen and lymph nodes of *Foxp3-hCD2* mice using an autoMACS Pro Separator with a CD4 isolation kit (Miltenyi Biotec) by negative selection (Depletes), and then positive selection (Possel_2) was achieved with anti-PE beads in the presence of PE-hCD2 (TS1/8). Then, the cells were sorted using PE-conjugated anti-hCD2, allophycocyanin (APC) anti-CD4 (RM4-5) and BV421 anti-CD45 (30-F11) antibodies with a Sony SH800 Cell Sorter (Sony Biotechnology). The cells were mixed with 100 μl of primary cell nucleofection solution (P4 Primary Cell 4D-Nucleofector X kit L; 24 reactions, V4XP-4024; Lonza). The cells were then incubated with 15 μl of RNP complex, transferred to a single nucleofection cuvette, and electroporated using a 4D nucleofector (Lonza) with a CV137 pulse. After nucleofection, the transfected cells were transferred to 96-well plates with prewarmed 200 μl of complete T cell medium and cultured for 30 min. After resting in culture, conventional T cells (4.0 × 10^6^ cells) and transfected Tregs (1 × 10^6^ cells) were transferred intravenously into *Cd3ε*^−/−^ mice.

### In vitro analysis

Tregs were isolated from spleens and LNs using an autoMACS Pro Separator with a CD4 isolation kit (Miltenyi Biotec) by negative selection (Depletes) and then sorted using PE-conjugated anti-CD25 (PC6.1), APC anti-CD4 (RM4-5) and BV421 anti-CD45 (30-F11) with a Sony SH800 Cell Sorter (Sony Biotechnology). Tregs (4 × 10^5^ cells) were cultured with plate-coated anti-CD3ε Ab (﻿145-2C11: 2 μg mL^−1^), anti-CD28 Ab (57.31; 1 μg ml^−1^) and IL-2 (10 ng ml^−1^: PeproTech) in RPMI 1640 supplemented with 10% FBS in 24-well culture dishes for 24 h. Live cells were counted with optical density measurements using Cell Count Reagent SF (Nacalai Tesque).

### Microarray analysis

With the exception of the kidney data, microarray analysis was based on the data from our previous paper.^7^ For CD45^+^CD3e^+^CD4^+^FoxP3-hCD2^+^ T cells from renal tissue, total RNA was isolated using TRIzol LS reagent (Invitrogen). Microarray analysis was performed by CERI (Chemical Evaluation and Research Institute, Japan) using SurePrint G3 Mouse Gene Expression 8 × 60 K v2 (Agilent Technologies). The expression values were calculated using feature extraction software (Agilent Technologies). The values were normalized by adjusting each set of expression data to the 75th percentile baseline following log_2_ transformation. (The normalization step was originally performed with the implementation of the 75th percentile normalization algorithm.) Further analyses were performed and graphics generated using ExAtlas^63^ and GeneSpring (Agilent) software, respectively. The microarray data of other non-renal tissue Tregs were obtained from the Gene Expression Omnibus database (VAT Tregs, GSE37532; injured muscle Tregs, GSE50096; and colon Tregs, GSE68009) and analyzed using ExAtlas following quantile normalization as described.^7^

### Quantitative PCR (qPCR)

mRNA was extracted from renal tissues using an RNAiso kit (TaKaRa Bio, Japan). Total RNA was reverse transcribed to cDNA using a high-capacity cDNA reverse transcription kit (Applied Biosystems). qPCR was performed on the cDNA samples using EvaGreen (Bio-Rad). The relative quantification value was expressed as 2^−ΔCt^, in which ΔCt is the difference between the mean Ct value of triplicate measurements and the endogenous GAPDH control.

### Statistics

Data are expressed as the means ± SEM. Statistical significance was determined by the Tukey–Kramer HSD test based on the differences among ≥3 groups, by unpaired two-sided Student’s *t*-test or two-sided Wilcoxon rank-sum test for differences between two groups, and by Wilcoxon signed-rank test between two paired groups. Pearson’s correlation coefficient was used to calculate the correlation analysis. *P* < 0.05 was considered a significant difference. All analyses were performed utilizing JMP 14 software (SAS Institute Inc.).

## Supplementary information

Supplemental figure

Supplemental table
