# Supplementary material for: Kidney GATA3+ regulatory T cells play roles in the convalescence stage after antibody-mediated renal injury
Source: Cell Mol Immunol. 2020 Sep 11;18(5):1249–61. doi: 10.1038/s41423-020-00547-x (PMC8093306; doi:10.1038/s41423-020-00547-x)
Supplement: Supplementary file 1 — Supplemental figure [file 41423_2020_547_MOESM1_ESM.pdf]

SUPPLEMENTAL FIGURE

For; R Sakai et al. **Kidney GATA3^{+} regulatory T cells play roles in convalescence stage after antibody-mediated renal injury**

Supplemental Figure. 1

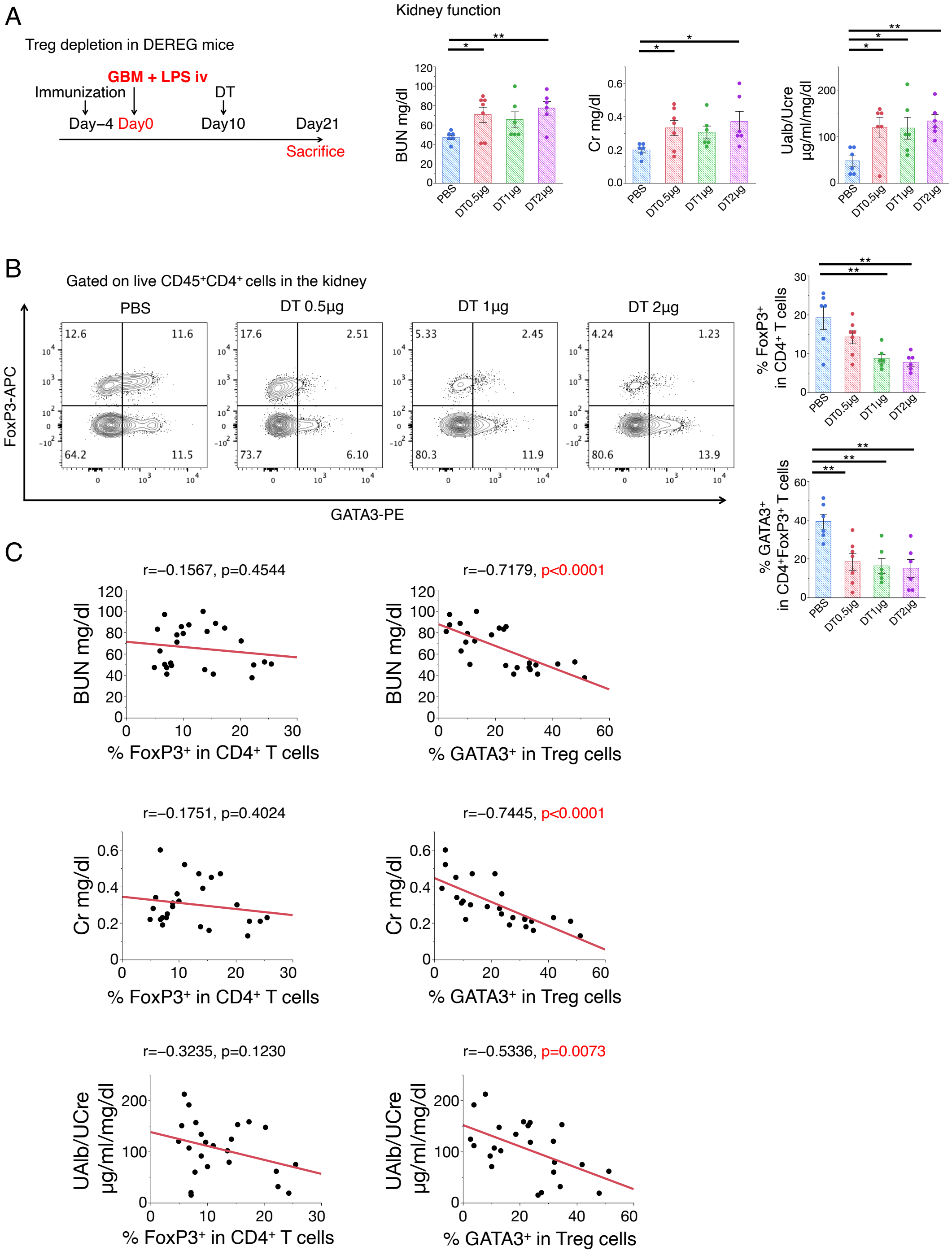

Supplemental Figure 1

(A) Schematic procedure for Treg depletion in DEREG mice. DEREG mice were injected intraperitoneally with indicated amount of diphtheria toxin (DT) on day 10 and analyzed on day 21. Kidney function was assessed by measuring serum BUN and creatinine levels and determining ratios of urine albumin (UAlb)/urine creatinine (UCre) (n = 6−7). (**B**) Representative dot plots of flow cytometry (FCM) analysis gated on $FVD^{-}CD45^{+}CD4^{+}$ isolated from the kidney of DEREG mice. The proportion of $FoxP3^{+}$ per $FVD^{-}CD45^{+}CD4^{+}$ cell and $GATA3^{+}$ cells per $FVD^{-}CD45^{+}CD4^{+}FoxP3^{+}$ cell in kidney tissues (n = 6−7). **(C)** Pearson's correlation of kidney function and the proportion of $FoxP3^{+}$ cell in $CD4^{+}$ T cells or the proportion of $GATA3^{+}$ cells in $CD4^{+}FoxP3^{+}$ cells in kidney. Symbols represent individual data points of mice, and the data were pooled from two independent experiments. The horizontal lines indicate mean ± SEM; $^{*}P < 0.05$ and $^{**}P < 0.01$ (Tukey–Kramer HSD test).

Supplemental Figure. 2

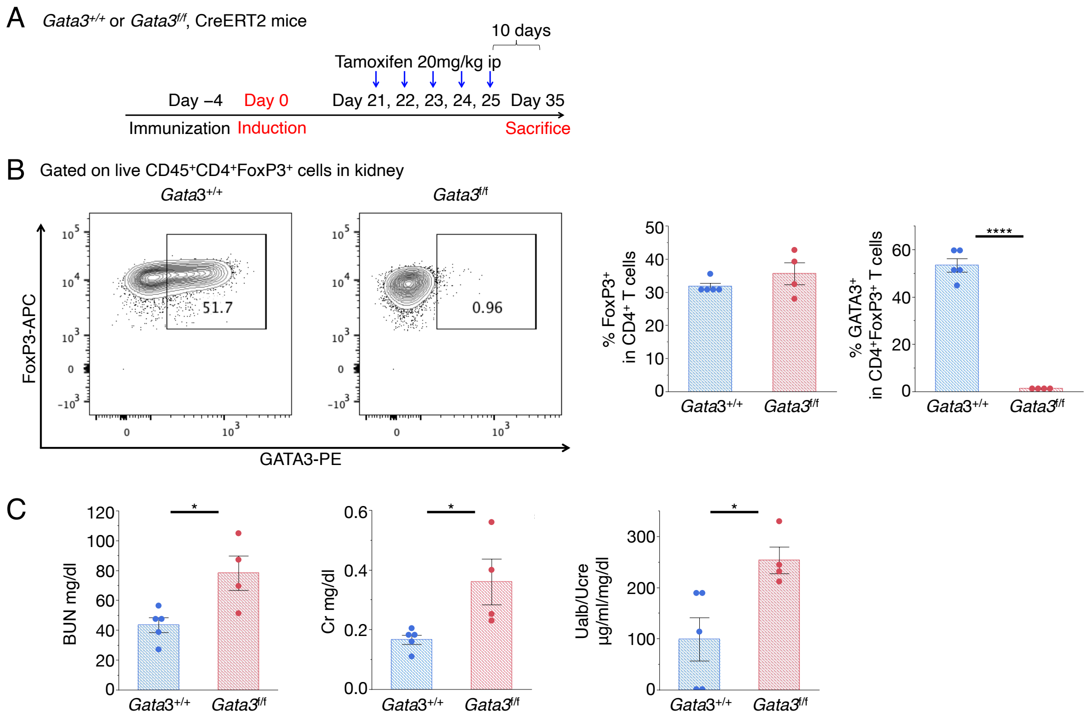

Supplemental Figure 2

(A) Schematic procedure for induction of cGN in *Gata3*$^{+/+}$ or *Gata3*$^{f/f}$, ERT2-Cre mice. **(B)** Representative dot plots of FCM analysis gated on FVD^{-}CD45^{+}CD4^{+}FoxP3^{+} isolated from the kidney of *Gata3*$^{+/+}$ or *Gata3*$^{f/f}$, ERT2-Cre mice. The proportion of FoxP3^{+} per FVD^{-}CD45^{+}CD4^{+} cell and GATA3^{+} cells per FVD^{-}CD45^{+}CD4^{+}FoxP3^{+} cell in kidney tissues (n = 4–5). (C) Kidney function was assessed by measuring serum BUN and creatinine levels and determining ratios of urine albumin (UAlb)/urine creatinine (UCre) (n = 4–5). Symbols represent individual data points of mice, and horizontal lines indicate means ± SEM; $^{*}P < 0.05$ and $^{****}P < 0.0001$ (Student's *t*-test).

Supplementary Figure. 3

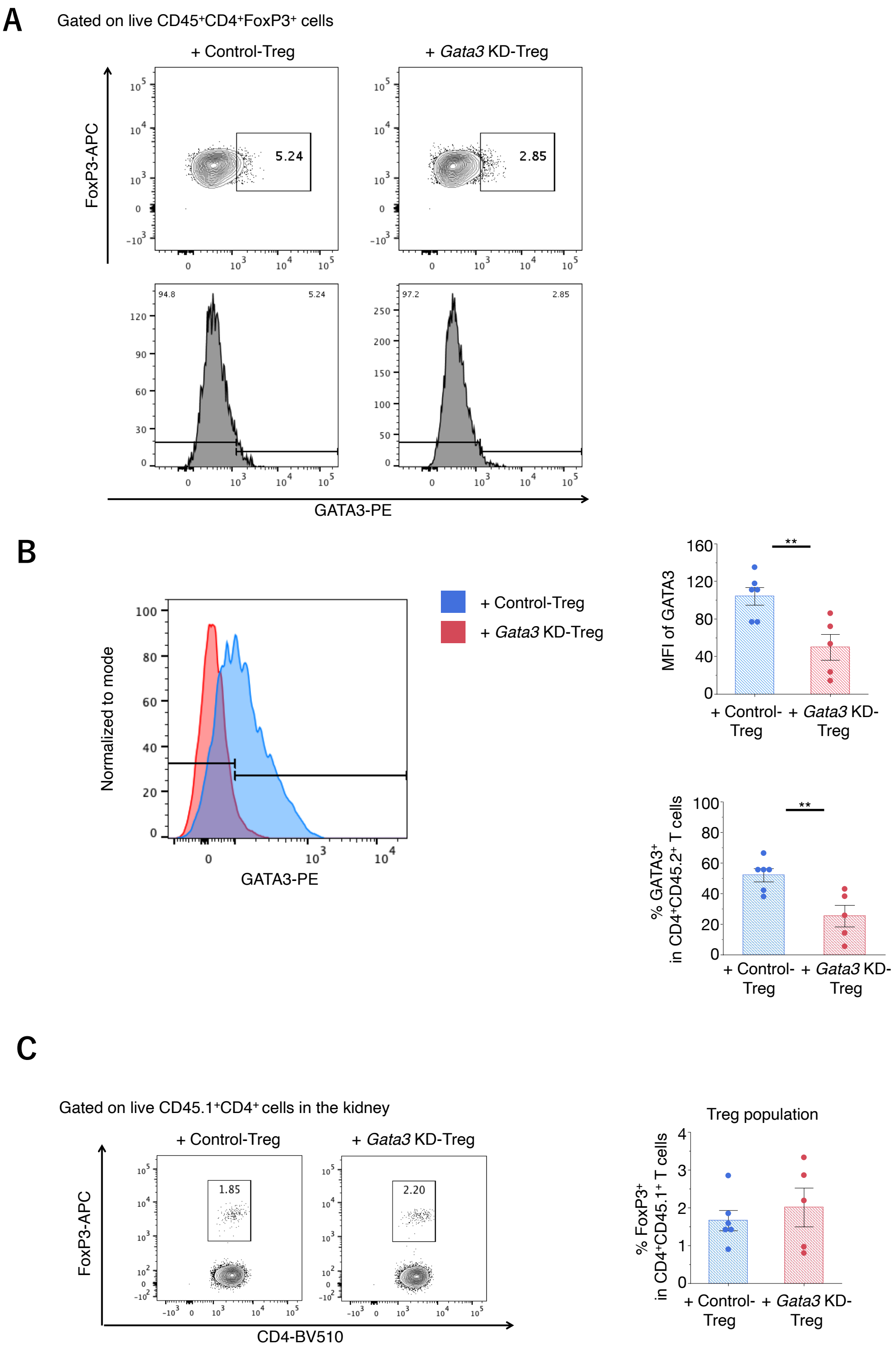

Supplemental Figure 3

Efficiency of *Gata3* knockdown by CRISPR/Cas9. **(A)** Representative dot plots of FCM analysis and histograms of GATA3 expression gated on $FVD^{-}CD45^{+}CD4^{+}FoxP3^{+}$ cells right after transduced by CRIPSR–Cas9 (control gRNA or *Gata3* gRNA). **(B)** Representative histograms of GATA3 expression gated on $FVD^{-}CD45^{+}CD4^{+}FoxP3^{+}$ isolated from the kidney of adoptive transferred $Cd3\varepsilon^{-/-}$ mice on day 28. Quantification of MFI of GATA3 and the proportion of $GATA3^{+}$ cells per $FVD^{-}CD45^{+}CD4^{+}FoxP3^{+}$ cell in kidney tissues (n = 5−6). (**C**) Representative dot plots of FCM analysis gated on $FVD^{-}CD45.1^{+}CD45.2^{-}CD4^{+}$ isolated from the kidney of adoptive transferred $Cd3\varepsilon^{-/-}$ mice on day 28 after cGN induction. The proportion of $FoxP3^{+}$ per $FVD^{-}CD45.1^{+}CD45.2^{-}CD4^{+}$ cell in kidney tissues (n = 5−6). Symbols represent individual data points of mice representative of two independent experiments, and horizontal lines indicate means ± SEM; $^{**}P < 0.01$ (Student's *t*-test).

Supplementary Figure. 4

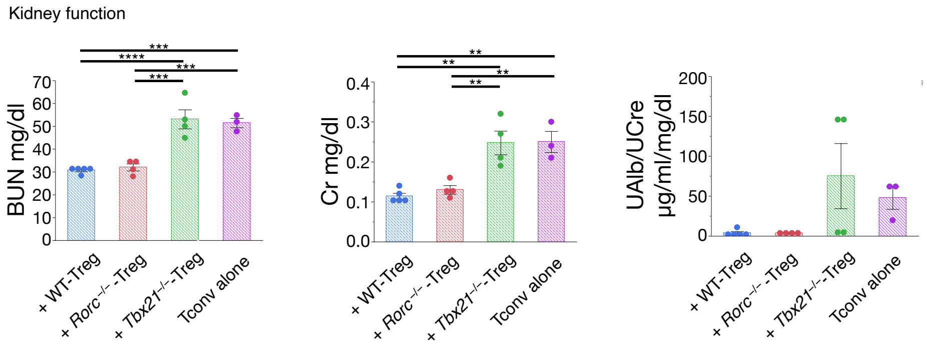

Supplemental Figure 4

Kidney function on day 28 after cGN induction was assessed by measuring serum BUN and creatinine levels and determining ratios of urine albumin (UAlb)/urine creatinine (UCre) from *Cd3ε*$^{-/-}$ mice by adoptive transferred of CD4^{+}CD25^{-} (Tconv: 4.0 × 10^{6}) cells with or without CD4^{+} CD25^{+} (Treg: 1.0 × 10^{6}) from spleens and lymph nodes in WT mice, *Rorc*$^{-/-}$ mice and *Tbx21*$^{-/-}$ mice (n = 3−5). Symbols represent individual data points of mice, and horizontal lines indicate means ± SEM; $^{**}P < 0.01$ and $^{***}P < 0.001$ (Tukey–Kramer HSD test).

Supplemental Figure. 5

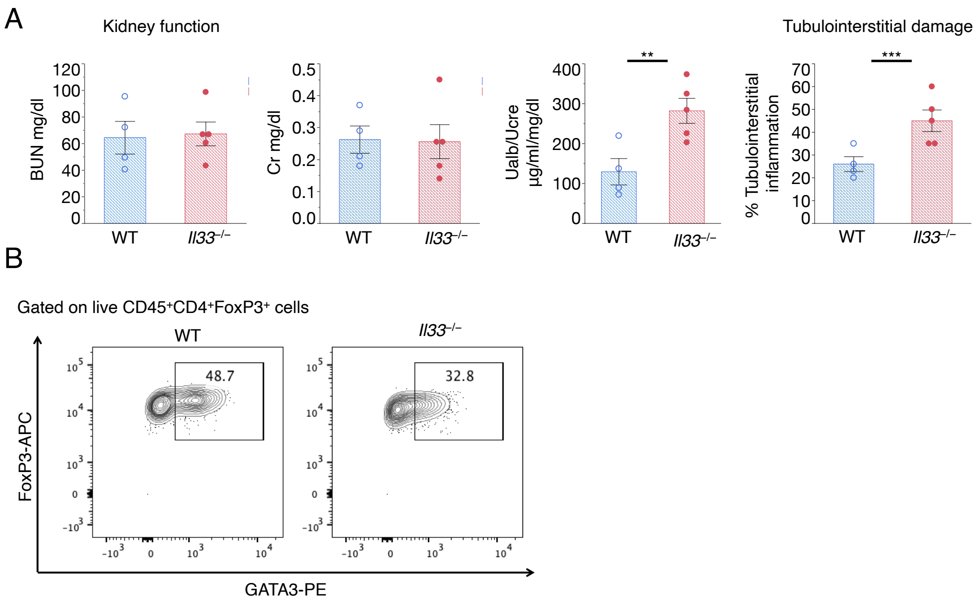

Supplemental Figure 5

(**A**) Analysis of *Il33*$^{-/-}$ mice sacrificed on day 56. Kidney function was assessed by measuring serum BUN and creatinine levels according to ratios of urine albumin (UAlb)/urine creatinine (UCre). Interstitial inflammation was evaluated according to renal pathological findings (n = 4–5). (**B**) Representative dot plots of FCM analysis gated on FVD^{-}CD45^{+}CD4^{+}FoxP3^{+} isolated from the kidney of WT and *Il33*$^{-/-}$ mice. Symbols represent individual data points of mice representative of two independent experiments, and horizontal lines indicate means ± SEM; $^{*}P < 0.05$ and $^{**}P < 0.01$ (Student’s *t*-test).

Supplemental Figure. 6

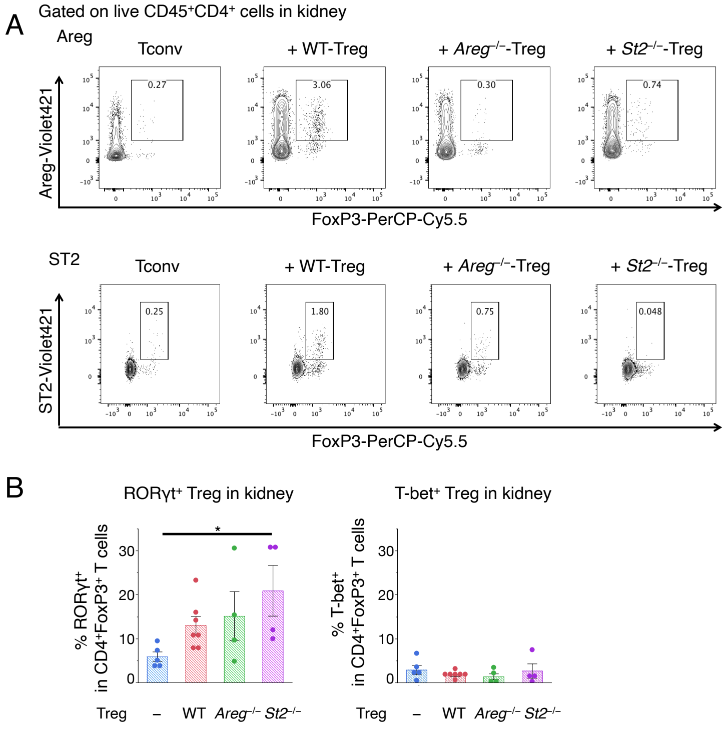

Supplemental Figure 6

(A) Representative dot plots of FCM analysis gated on per FVD$^-$CD45$^+$ CD4$^+$ cells in the kidney on day 28. The proportion of Areg$^+$ cells (upper panels) and ST2$^+$ cells (lower panels) per FVD$^-$FoxP3$^+$CD45$^+$CD4$^+$ cell in the kidney of adoptive transferred *Cd3ε*$^{-/-}$ mice. (**B**) The proportion of RORγt$^+$ cells (left) and T-bet$^+$ cells (right) per FVD$^-$CD45$^+$CD4$^+$FoxP3$^+$ cell in the kidney (n = 4−7). Symbols represent individual data points of mice representative of two independent experiments, and horizontal lines indicate means ± SEM; $^*P < 0.05$ (Tukey–Kramer HSD test).

Supplementary Figure. 7

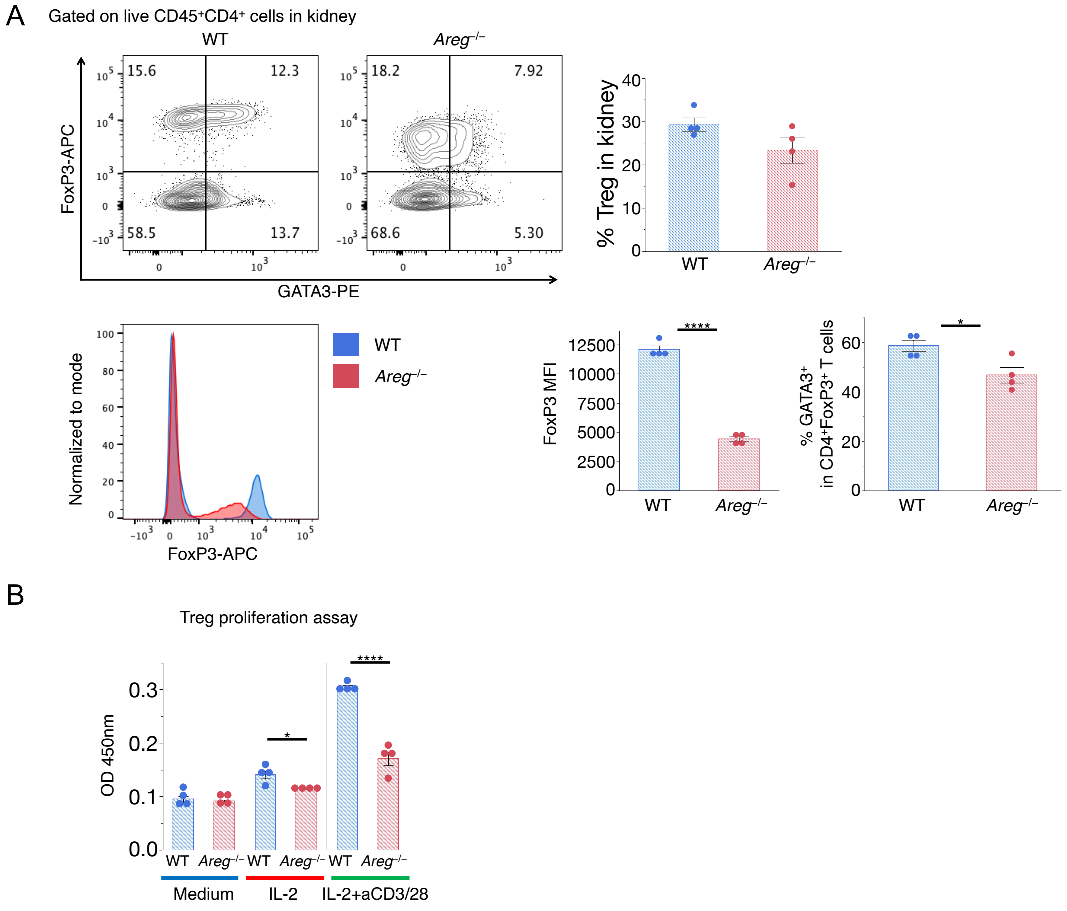

Supple mental Figure 7

(A) Representative dot plots of FCM analysis and histograms of FoxP3 gated on FVD$^-$CD45$^+$CD4$^+$ isolated from the kidney of WT and *Areg*$^{-/-}$ mice on day 28 after cGN induction. The proportion of FoxP3$^+$ per FVD$^-$CD45$^+$CD4$^+$ cell, GATA3$^+$ cells per FVD$^-$CD45$^+$CD4$^+$FoxP3$^+$ cell in kidney tissues and quantification of MFI of FoxP3 (n = 4). Symbols represent individual data points of mice representative of two independent experiments, and horizontal lines indicate means ± SEM; $^{*}P < 0.05$ and $^{****}P < 0.0001$ (Student's *t*-test). **(B)** Tregs (4 × 10^5 cells) were cultured with plate-coated anti-CD3ε Ab (2 μg/mL), anti-CD28 Ab (1 μg/ml) and IL-2 (10 ng/ml) in 24-well culture dish for 24 h. Live cells was counted with optical density measurements (OD) using Cell Count Reagent (n = 4). Symbols represent individual data points

representative of two independent experiments, and horizontal lines indicate means ± SEM; $^{*}P < 0.05$ and $^{****}P < 0.0001$ (Student's *t*-test).

Supplemental Figure. 8

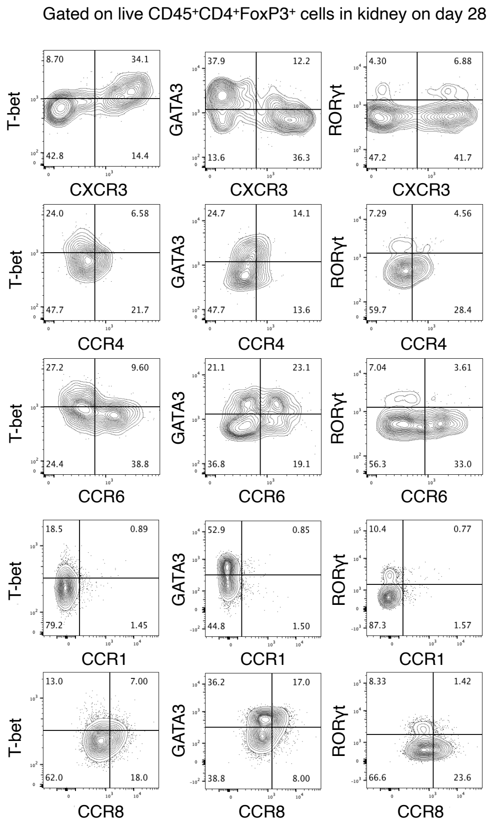

Supplemental Figure 8

Representative dot plots of FCM analysis of RORγt, GATA3, T-bet, and various chemokine receptors gated on $FVD^{-}CD45^{+}CD4^{+}FoxP3^{+}$ cells from kidneys on day 28.

Supplementary Figure. 9

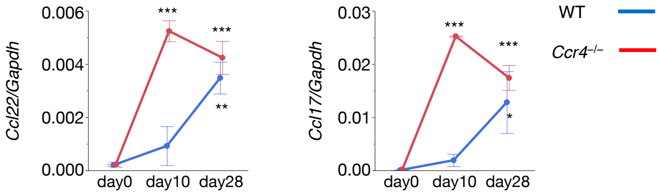

Supplemental Figure 9

Ccl22/17 mRNA expression levels were normalized to those of *Gapdh* in kidney tissues in WT or *Ccr4*$^{-/-}$ mice on day 0, day 10 and day 28. Symbols represent mean data points of mice representative of two independent experiments, and horizontal lines indicate means ± SEM; $^{*}P < 0.05$, $^{**}P < 0.01$ and $^{***}P < 0.001$ (Tukey–Kramer HSD test).

Supplementary Table 1

All antibodies and reagents are described in another file.
